# Has the Germ Oe Yellow Fever Been Found?

**Published:** 1899-09

**Authors:** 


### TEXAS MEDICAL JOURNAL.

AUSTIN, TEXAS.

A MONTHLY JOURNAL OF MEDICINE AND SURGERY.

F. E. DANIEL, M. D., Editor.

Published Monthly at Austin, Texas, by Drs. Daniel and Hudson. Subscription price $1.00 a year in advance.

Eastern Representative: John Guy Monihan, St. Paul Building, 220 Broadway, New York City.

Official organ of the West Texas Medical Association, the Houston District Medical Association, the Austin District Medical Society, the Brazos Valley Medical Association, the Galveston County Medical Society, and several others.

### HAS THE GERM OE YELLOW FEVER BEEN FOUND?

Bearing in mind the false alarms of Domingoes-Friere, Carmona y Valle, and perhaps others, who some time ago confidently proclaimed the discovery of the yellow fever bacillus, the medical profession are slow to believe the claims of others to the discovery, and look upon all such claims with suspicion. At the present writing things look favorable to establishing the validity of Sanarelli's claim, and cultures of the alleged *bacillus icteroides* have been made, serum prepared and used, it is claimed, with success on the human subject. Dr. Doty, of New York, reports the case of a Mr. Lackey who recovered from yellow fever under the use of a serum so prepared, and priority has been claimed for Dr. Doty in such treatment. According to the *New Orleans Medical and Surgical Journal* (August, 1899), Dr. Archinard, of New Orleans, as far back as September, 1898, treated twelve cases of yellow fever in the Charity Hospital at New Orleans, with a Sanarelli serum, sent him from Montevideo, with eight recoveries; but it seems that Dr. Archinard does not give the serum credit for the recoveries, but says that the patients would have probably recovered without it. Again, Drs. Wasdin and Geddings, of the Marine Hospital Service, who were appointed by the secretary of the treasury and the president to go to Havana and investigate the subject, report that the Sanarelli bacillus is the undoubted cause of the disease and that its antitoxine is the correct thing, and a serum treatment will fill the indications. Per contra, the bacteriologists of the army medical museum at Washington, under Surgeon General Sternburg, say that Sanarelli's microbe is a species of the bacillus of hog cholera; that it is occasionally and accidentally found in yellow fever patients, but that "its etiological relations to that disease has not been established."

